# Patterns of Brain Activation when Mothers View Their Own Child and Dog: An fMRI Study

**DOI:** 10.1371/journal.pone.0107205

**Published:** 2014-10-03

**Authors:** Luke E. Stoeckel, Lori S. Palley, Randy L. Gollub, Steven M. Niemi, Anne Eden Evins

**Affiliations:** 1 Department of Psychiatry, Massachusetts General Hospital, Boston, Massachusetts, United States of America; 2 Center for Comparative Medicine, Massachusetts General Hospital, Boston, Massachusetts, United States of America; 3 Department of Psychiatry, Harvard Medical School, Boston, Massachusetts, United States of America; 4 Faculty of Arts and Sciences, Harvard University, Cambridge, Massachusetts, United States of America; University of Tuebingen Medical School, Germany

## Abstract

Neural substrates underlying the human-pet relationship are largely unknown. We examined fMRI brain activation patterns as mothers viewed images of their own child and dog and an unfamiliar child and dog. There was a common network of brain regions involved in emotion, reward, affiliation, visual processing and social cognition when mothers viewed images of both their child and dog. Viewing images of their child resulted in brain activity in the midbrain (ventral tegmental area/substantia nigra involved in reward/affiliation), while a more posterior cortical brain activation pattern involving fusiform gyrus (visual processing of faces and social cognition) characterized a mother's response to her dog. Mothers also rated images of their child and dog as eliciting similar levels of excitement (arousal) and pleasantness (valence), although the difference in the own vs. unfamiliar child comparison was larger than the own vs. unfamiliar dog comparison for arousal. Valence ratings of their dog were also positively correlated with ratings of the attachment to their dog. Although there are similarities in the perceived emotional experience and brain function associated with the mother-child and mother-dog bond, there are also key differences that may reflect variance in the evolutionary course and function of these relationships.

## Introduction

Humans began domesticating dogs to serve in a variety of roles, including as human companions or ‘pets’, 18,000–32,000 years ago [1]. The practice of adopting and nurturing other species (like dogs) or “alloparenting” is a common human behavior across different cultures that arose from the evolutionary need for domestication [2]. Approximately 2/3 of U.S. households have pets, and over $50 billion is spent annually on their care (http://www.americanpetproducts.org/press_industrytrends.asp). Many people have a strong emotional attachment to their pets. Pet owners have been termed ‘pet parents’ in the popular media, and half of pet owners consider their pet as much a part of the family as any member of the household (AP-Petside.com Poll 2009). Pets can be beneficial to the physical, social, and emotional well-being of humans [3]–[6], and animal-assisted therapy is widely used as a complementary medicine and adjunctive mental health intervention [7], [8].

Similarities between the owner-dog relationship and the human-infant relationship have been described within the framework of human attachment theory, developed to explain the role of the human infant-caregiver relationship in development, and extended to adult-adult caregiver, peer, and romantic relationships [9]. Attachment, usually refers to the bond formed between a child and caregiver (typically, a mother) to ensure safety, security, and, ultimately, survival [10] that may apply also to the formation and maintenance of people's relationship with their pets [11]–[13].

On a well-established laboratory-based infant-maternal attachment measure [14], [15], very similar results for human infants' and dogs' behaviors with their mother or owner have been described under high and low stress conditions [15]–[17]. Similar neurobiologic mechanisms of bonding have been implicated in human-human and owner-dog pairs. Oxytocin, beta-endorphin, prolactin, beta-phenylethylamine, and dopamine are increased in pet owners and their dogs during [18] and after [19]–[21] a positive interaction.

Functional magnetic resonance imaging (fMRI) has been used to investigate neural responses when humans view the faces of their romantic partner or child compared with other faces [22]–[24]. Some brain regions activated to objects of both maternal and romantic love overlap with the brain's reward system that is hypothesized to facilitate strong interpersonal attachments [22]. Some common regions of activation also have dense expression of oxytocin and vasopressin receptors implicated in pair-bonding and maternal attachment [23].

In this study, our aim was to directly compare the functional neuroanatomy of the human-pet bond with that of the maternal-child bond. To do so, we analyzed patterns of brain function when mothers viewed images of their own child and own dog, with the aim of discovering both distinct and common regions of activation. We focused our analyses on specific brain regions of interest (ROI) known to be involved in the formation and maintenance of social bonds.

## Methods

**Figure 1:**
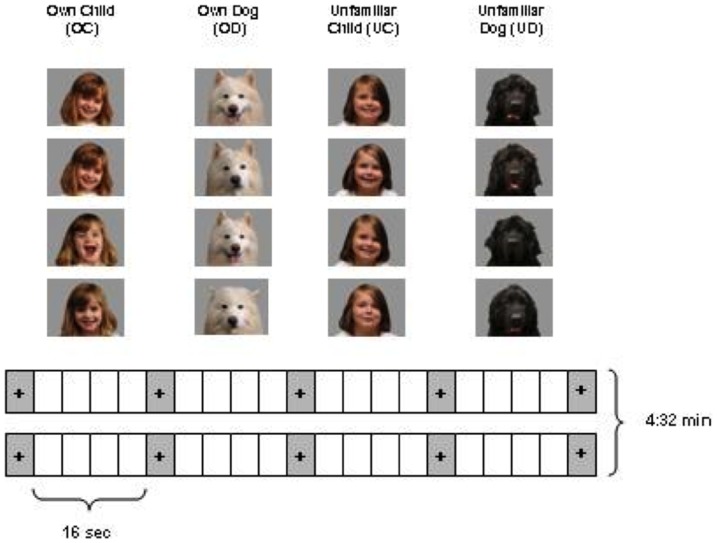
Study Schematic of the Experimental Design. Illustration of the passive viewing paradigm of dog and child images used. Sixteen unique color photos of faces: 4 own child (OC), 4 own dog (OD), 4 unfamiliar child (UC), 4 unfamiliar dog (UD) presented in 16 sec blocks (4 images/block) over 6 fMRI runs. Each block of images was followed by a screen with a fixation cross (FX).

The study was approved by the Partners Human Research Committee. Participants provided full written informed consent prior to beginning study procedures. The individuals in this manuscript have given written informed consent (as outlined in the PLOS consent form) to publish the images of their child's and dog's face (Figure 1) and other case details.

### Participants

Participants were recruited via advertisement in local media, veterinary clinics, dog parks, and the Massachusetts General Hospital Research Study Volunteer Program for Health Registry. Eligible participants were women, aged 22–45 years, who had at least one child, aged: 2–10 years, and one pet dog, owned for at least 2 years, reported low to normal parenting stress (total score <90 on the Parenting Stress Index-Short Form (PSI-SF) [25]), normal affect (positive affect >12.5 and negative affect <29.1 on the Positive and Negative Affect Scale; PANAS [26], were right-handed, and had at least average estimated intellectual function (estimated Full Scale IQ >85 on Weschler Test of Adult Reading (WTAR); [27]. Exclusion criteria included any self-reported lifetime Axis I psychiatric disorder, current major medical illness, conditions that may impact brain reward function (e.g., obesity, substance use, pathological gambling), current or planned pregnancy, use of CNS-active medication in the prior six months, contraindication to MRI, and working in an animal-related field.

### Assessments

#### Study Session 1 (home visit)

Participants' child and dog were photographed in the participants' home, and participants completed the Edinburgh Handedness Inventory [28], PSI-SF [25], WTAR [27], the PANAS [26], Lexington Attachment to Pets Scale (LAPS; [29], and a demographic and dog ownership questionnaire. Participants were then shown a series of unfamiliar child and dog photographs, assembled from participants who consented to having photographs of their child and dog viewed by others in the study, and were asked, “Are you familiar with this child or dog?” to confirm that control images were “unfamiliar”.

*Visual stimuli preparation*: Sixteen unique photographs of children and dogs were selected and edited for each participant in Adobe Photoshop Elements 8.0. The unfamiliar child and dog images were selected based on the familiarity assessment, and the unfamiliar child images were matched to the participant's child for gender and age. Photographs were cropped to 4×3 inches (to include the whole face with minimal neck and shoulders), resized to 800×600 pixels, outlined, and the selected area outside the image was shaded neutral grey. Images were converted to bitmap (*.bmp) format and modified for consistent luminance.

#### Study Session 2 (imaging visit)

Participants completed the PANAS and were then placed in the MRI scanner. They received instructions to relax as they passively viewed a variety of images of children and dogs (including some photographs taken during their home visit) as well as a fixation cross. Immediately following the scanning session, participants were given an eleven-question, multiple choice recognition test of the images they viewed in the scanner to verify that they were attentive during the study. Participants were asked about the content of the images, the hair color of the children and dogs, the number of images displayed, etc. Participants were then asked to rate 5 images per category selected from those shown during the scanning session on their emotional value (valence or pleasantness and arousal or excitement; [30]) using the Self Assessment Manikin scale (SAM; [31]).

*MRI data acquisition and procedure*: Brain imaging data were acquired on a 3 Tesla Siemens TIM Trio MRI scanner using a 32-channel head coil. Blood-oxygen-level-dependent (BOLD) functional MRI data were acquired using a gradient echo T2*-weighted pulse sequence (TR/TE  = 2000/30 ms, flip angle  = 90°, FOV  = 200×200 mm, 32 axial oblique slices collected −30 degrees off the AC-PC line, slice thickness  = 3.0 mm with 0.3 mm interslice gap, 816 image volumes per slice, matrix  = 64×64). A high-resolution 3D MPRAGE sequence was collected for anatomic localization of the fMRI data. For the fMRI scans, visual stimuli (photographs) were presented to participants in a block design format, with six 4∶32 min runs per imaging session. Each run consisted of two 16 s epochs each for each image category. Within each 16 s epoch of images, four individual images were presented for 3.5 s each. A 0.5 s gap separated the images, and a pseudorandom gap of 14, 16, or 18 s separated the epochs. All gaps consisted of a gray blank screen with a fixation cross (Fig. 1). Each run consisted of 136 volumes for a total of 816 volumes across six runs, of which 96 volumes were acquired for each image category. The visual images were presented with a Windows XP laptop computer running PsychToolbox (http://psychtoolbox.org/HomePage) and a Matlab (Mathworks, Inc., 2000) toolbox. Images were projected onto a screen behind the participant's head at the back of the scanner and viewed via a 45° single-surface rear-projecting mirror attached to the head coil. Eye movements were not monitored during imaging, as emotional and neutral images have been reported to result in no differential eye movements [32], [33].

*fMRI analysis*: fMRI data analysis was conducted with Statistical Parametric Mapping, Version 8 (SPM8: http://www.fil.ion.ucl.ac.uk/spm/software/spm8/) and custom Matlab routines. Standard image preprocessing was performed including motion and field map distortion correction, normalization to the Montreal Neurological Institute (MNI) standard brain template space, and spatial smoothing with a 6 mm FWHM Gaussian filter. Artifact detection and removal was performed using ART (http://web.mit.edu/swg/software.htm). Specifically, an image was defined as an outlier (artifact) image if the head displacement in x, y, or z direction was greater than .5 mm from the previous frame, or if the rotational displacement was greater than .02 radians from the previous frame, or if the global mean intensity in the image was greater than 3 standard deviations from the mean image intensity for the entire resting scan. There were five outliers total across the 14 participants (2 during the own child images and 3 during the fixation period).

Preprocessed block design BOLD fMRI data were analyzed in normalized (MNI) space within the context of the General Linear Model on a voxel-by-voxel basis as implemented in SPM8. The time course of brain activation was modeled with a boxcar function convolved with the canonical hemodynamic response function (HRF), including a temporal derivative function. Individual regressors included task conditions, six motion parameters (3 translational and 3 rotational directions), and outliers (one regressor per outlier image identified with ART). A two-stage procedure was used for the statistical analysis of a mixed-effects design in SPM8 [34]. We analyzed the data using a 2×2 repeated measures analysis of variance (ANOVA) to assess the main effects of species (child vs. dog), relationship (own vs. unfamiliar), and the species x relationship interaction using the flexible factorial approach in SPM8. We then generated statistical contrasts for comparing brain activation in response to 1) own child vs. fixation, 2) own dog vs. fixation, 3) own child vs. own dog, 4) own child vs. unfamiliar child, and 5) own dog vs. unfamiliar dog using planned one-sample t-tests. To address our *a priori* hypotheses and to improve statistical power, we used a ROI approach and small volume correction (SVC) in SPM8 [35]. Briefly, SVC is a voxelwise approach controlling the statistical threshold by only correcting for the number of voxels in the specified ROI(s). The size of the ROI masks used in the present study ranged from 104 mm^3^ or 13 voxels (HYPO) to 16,984 mm^3^ or 2,123 voxels (insula). Given the range in size in our ROIs and the potential for functional heterogeneity within these ROI masks, we chose the SVC approach as it would allow us to detect activation in a subset of voxels within these ROI masks. By averaging across the entire ROI mask, we may have less sensitivity to detect activation creating a bias towards the null [36].

*Brain regions (ROIs)*: Our regions of interest were based on previous fMRI studies in the literature implicating these regions in the neurobiology of the maternal-child relationship and facial perception [23], [37]–[39]. These included regions of the classic mesocorticolimbic dopamine reward/motivation system (ventral tegmental area (VTA), ventral striatum/nucleus accumbens (NAcc), amygdala, and medial orbitofrontal cortex (mOFC)), midbrain structures with dense expression of oxytocin and vasopressin receptors (substantia nigra (SNi) and periaqueductal grey (PAG)), and structures involved in social cognition and visual perception (superior temporal and fusiform gyri) and salience and interoceptive function (insula). Also included from these fMRI studies, were the hippocampus (HIPPO), hypothalamus (HYPO), thalamus, and dorsal striatum (caudate and putamen). ROI's were defined using anatomical structures in MNI space selected within the WFU Pickatlas toolbox [40] and the Harvard-Oxford atlas (http://fsl.fmrib.ox.ac.uk/fsl/fslwiki/Atlases). Regions unavailable in these libraries (VTA/Sn and PAG) were drawn within the WFU Pickatlas using 3 mm volume-based spheres centered at a voxel location as identified by previous studies (VTA/Sn: x = ±4, y = −14, z = −16 [23], [38]; PAG: x = ±2, y = −32, z = −24 [23]).

Significance for these *a priori* ROIs was assessed with cluster thresholds of *p*< .01 at the voxel level (uncorrected) and a familywise error (FWE) correction (as implemented in SPM8, using Gaussian Random Field Theory) of *p*< .05 at the cluster level. For the own child and own dog vs. fixation contrasts, we performed a conjunction analysis using the minimum statistic for conjunction null method [41], resulting in an overall alpha of *p*< .001 to determine whether shared brain regions were activated to both the own child and own dog images.

*Behavioral analyses*: Valence and arousal ratings of the own and unfamiliar dog and child images were analyzed with a 2 (child vs. dog) ×2 (own vs. unfamiliar) repeated measures ANOVA. Pearson product-moment correlations were calculated to test the association between mean valence and arousal ratings for the own and unfamiliar dog images and LAPS total score. Analyses were performed with SPSS Version 21.0 (SPSS 21, IBM Corp. Released 2012. IBM SPSS Statistics for Mac, Version 21.0. Armonk, NY: IBM Corp.).

## Results

Eighteen participants were enrolled and completed the home visit, 16 completed the MRI visit, and 14 had high quality fMRI data and were included in the analyses. See Table 1 for participant characteristics.

**Table 1:** Participant Characteristics (n = 14).

**Age** (mean years (SD; range))	38.4 (5.0; 28–44)
**Race** (Caucasian/No Response)	12/2
**Education** (mean years (SD; range))	16.4 (1.6; 14–18)
**IQ** (mean (SD; range))	110.4 (5.2; 99–117)
**LAPS** (mean (SD; range))	48.6 (6.3; 34–59)
**Marital Status** (married/divorced)	10/4
**Employment** (full-time, part-time, housewife, student)	8/3/2/1
**Child Gender** (male/female)	3/11
**Child Age** (mean years (SD; range))	5.3 (3.0; 2–10)
**Dog Gender** (male/female)	8/6
**Dog Age** (mean years (SD; range))	6.2 (2.5; 3–10.5)

IQ  =  Weschler Test of Adult Reading Full Scale IQ.

LAPS  =  Lexington Attachment to Pets Scale (higher score means greater level of attachment).

### fMRI Results

ANOVA resulted in a main effect for relationship (own vs. unfamiliar) in brain regions involved in emotion, reward, and affiliative processes (amygdala, PAG, SNi/VTA), salience/interoception (insula), and in associated structures (thalamus), including those involved in visual processing and social cognition (fusiform and superior temporal gyri) with greater brain activation for own than other child and dog (Table 2). There was no main effect of species (child vs. dog) or relationship x species interaction for any ROIs (all *ps* > .05).

**Table 2:** fMRI results for main effect of relationship (own vs. unfamiliar) in brain regions of interest.

ROI Analysis	Neurosynth^a^	Hem^b^	Cluster^c^	x^d^	y^d^	z^d^	F	*p*, FWE corrected^e^
Amygdala	Emotion	L	90	−24	2	−16	16.48	0.017
Fusiform Gyrus	Semantic	L	228	−50	−64	−18	16.08	0.009
	Face	R	254	50	−60	−16	28.73	0.006
Insula	Sensory/Image	L	193	−32	14	2	24.30	0.016
PAG	N/A	L	12	−2	−32	−22	12.89	0.035
SNi/VTA	Reward	L	19	−2	−16	−16	23.72	0.028
	Reward	R	18	2	−16	−16	17.22	0.029
STG	Emotion	L	359	−38	10	−12	26.11	0.003
Thalamus	Motor/Sensory	L	219	−10	−14	6	13.47	0.005
	Motor	R	235	6	−20	6	13.25	0.004

a Neurosynth term/function (www.neurosynth.org); N/A  =  not available in the Neurosynth atlas.

b Hemisphere: R, right, L, left

c Cluster size; number of contiguous voxels with p< 0.01.

d x, y, and z coordinates in MNI space.

e Familywise error corrected at the cluster level.

All results significant at *p*< .05, cluster-level family-wise error correction.

Follow-up t-tests revealed all significant main effects were the result of greater brain activation in the own vs. unfamiliar (own > unfamiliar) contrast. There were no significant differences in ROI activation for the unfamiliar > own contrast.

**Figure 2:**
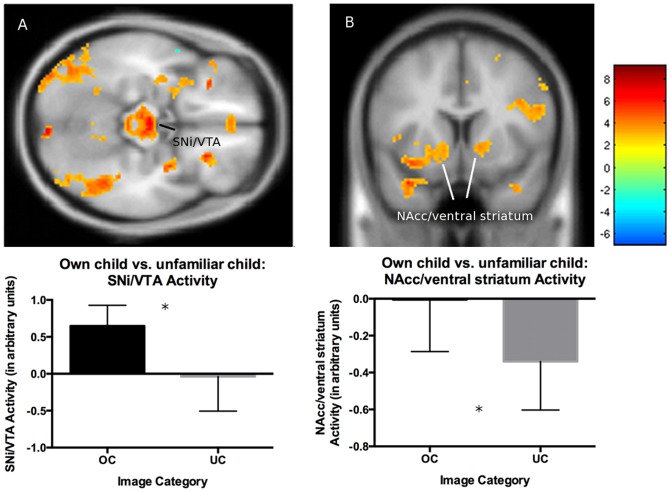
Brain activation maps and graphs for the own child (OC) vs. unfamiliar child (UC) contrast in mothers who are dog owners (n = 14). There was greater activation for the own child vs. unfamiliar child contrast in SNi/VTA (A) and less deactivation for this contrast in NAcc/ventral striatum (B). Bar graphs display ROI activation magnitude by image category using REX (http://web.mit.edu/swg/software.htm) to extract the beta/contrast values from significant clusters based on the results of our group-level fMRI analysis. Activation is overlaid on the SPM8 MNI 152 T1 template. Scale bar indicates t values. Error bars depict 95% confidence intervals.

Comparing BOLD activity when mothers viewed the own child vs. unfamiliar child images, mothers displayed increased activation in regions involved in reward and affiliation (SNi/VTA; Figure 2a) and associated structures (dorsal putamen, thalamus), including those involved in visual processing and social cognition (fusiform gyrus; Table 3). Additionally, the own child vs. unfamiliar child contrast elicited less *deactivation* in regions involved in reward and affiliation (NAcc/ventral striatum (Figure 2b): *t*(13)  = 3.13, *p* =  .041, cluster extent  = 28, MNI coordinates: x = −12, y = 10, z = −8; PAG: *t*(13) = 4.21, *p* =  .032, cluster extent  = 15, MNI coordinates: x = −2, y = −30, z = −22), salience/interoception (insula: *t*(13)  = 6.74, *p* =  .037, cluster extent  = 125, MNI coordinates: x = −32, y = 18, z = 8), and social cognition (superior temporal gyrus: *t*(13)  = 5.68, *p* =  .025, cluster extent  = 182, MNI coordinates: x = −36, y = 10, z = −26). There were no differences in any ROIs when comparing the own dog vs. unfamiliar dog images.

**Table 3:** fMRI results for own child > unfamiliar child contrast in brain regions of interest (n = 14).

ROI Analysis	Neurosynth^a^	Hem^b^	Cluster^c^	x^d^	y^d^	z^d^	t	*p*, FWE corrected^e^
Fusiform Gyrus	Face	R	262	40	−40	−18	6.67	0.003
Putamen	Face/Emotion	L	181	−24	−4	−8	4.22	0.006
SNi/VTA	Reward	L	17	−4	−16	−14	6.49	0.030
	Reward	R	18	2	−16	−16	6.06	0.029
Thalamus	Motor	L	180	−18	−16	10	3.94	0.005
	Planning	R	282	12	−10	8	4.66	0.001

a Neurosynth term/function (www.neurosynth.org).

b Hemisphere: R, right, L, left.

c Cluster size; number of contiguous voxels with p<0.01.

d x, y, and z coordinates in MNI space.

e Familywise error corrected at the cluster level.

All results significant at *p*< .05, cluster-level family-wise error correction.

There were no significant differences for the own dog vs. unfamiliar dog contrast.

**Figure 3:**
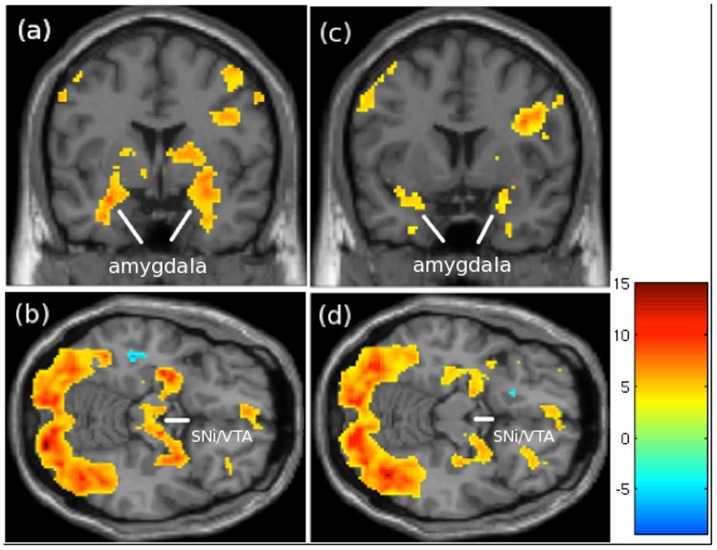
Greater activation for own child (OC) vs. fixation cross (FX; a,b) and own dog (OD) vs. FX (c,d) contrasts in mothers who are dog owners (n = 14). Note the more extensive activation in (a) amygdala (coronal view) for the OC > FX compared to the OD > FX images (c). There is also activation in (b) SNi/VTA (axial view) for the OC > FX images that is not present in the OD > FX images (d). Activation is overlaid on SPM8 single subject T1 template. Other conventions the same as in Figure 2.

Although we did not observe a main effect of species, we had *a priori* hypotheses about the mother's expected brain activation patterns in response to their own child and own dog images; therefore, we also tested the own child and own dog (vs. fixation) and own child vs. own dog contrasts. There were largely overlapping areas of increased BOLD activity when mothers viewed their own child or own dog vs. fixation screen in brain regions involved in emotion, reward, affiliative (amygdala) and associated functions (hippocampus, med OFC, dorsal putamen, thalamus), including visual processing and social cognition (fusiform gyrus) (Tables 4,5; Figure 3). Images of own child, but *not* own dog vs. fixation, activated additional regions involved in reward function (SNi/VTA). There were *no* brain regions active when viewing the own dog images that were not also activated by the own child images.

**Table 4:** fMRI results for the own child > fixation contrast in brain regions of interest (n = 14).

ROI Analysis	NeuroSynth^a^	Hem^b^	Cluster^c^	x^d^	y^d^	z^d^	t	*p*, FWE corrected^e^
Amygdala	Emotion	L	155	−28	−4	−14	10.23	<0.001
	Emotion	R	198	30	0	−16	9.65	<0.001
Fusiform Gyrus	Visual/Face	L	558	−42	−54	−18	9.84	<0.001
	Visual/Object	R	770	44	−70	−18	9.69	<0.001
Hippocampus	Memory	R	119	24	−24	−8	9.02	<0.001
Med OFC	Self/Valence	L	70	−2	48	−16	6.40	0.001
	Social/Valence	R	38	2	42	−20	4.87	0.006
Putamen	Facial	L	123	−24	−4	−8	6.78	<0.001
	Planning	R	325	32	4	−6	8.24	<0.001
SNi/VTA	Reward	L	13	−4	−12	−14	5.78	0.003
	Reward	R	5	6	−14	−14	4.49	0.006
Thalamus	Memory	L	74	−22	−30	−2	8.10	<0.001
	Motor	L	39	−12	−16	0	7.39	0.003
	Memory	R	99	24	−28	−2	9.59	<0.001
	Sensory/Motor	R	159	12	−12	4	7.69	<0.001

a Neurosynth term/function (www.neurosynth.org).

b Hemisphere: R, right, L, left.

c Cluster size; number of contiguous voxels with p<0.05.

d x, y, and z coordinates in MNI space.

e Familywise error corrected at the cluster level.

All results significant at *p*< .05, cluster-level family-wise error correction.

**Table 5:** fMRI results for the own dog > fixation contrast in brain regions of interest (n = 14).

ROI Analysis	Neurosynth^a^	Hem^b^	Cluster^c^	x^d^	y^d^	z^d^	t	*p*, FWE corrected^e^
Amygdala	Face/Emotion	L	170	−30	0	−20	6.87	<0.001
	Emotion	R	150	22	−2	−16	6.91	<0.001
Fusiform Gyrus	Visual	L	757	−42	−56	−20	9.34	<0.001
	Visual/Face	R	860	42	−66	−18	12.85	<0.001
Hippocampus	Memory	L	320	−20	−28	−6	9.25	<0.001
	Memory	R	226	24	−30	−4	8.76	<0.001
Med OFC	Social/Valence	L	164	0	40	−20	9.02	<0.001
	Affect	R	102	2	40	−20	7.28	<0.001
Putamen	Sensory/Motor	L	62	−24	−10	10	7.82	0.001
	Motor	L	25	−28	−16	−2	5.78	0.012
	Salience	R	95	30	−8	−8	8.31	<0.001
Thalamus	Memory	L	85	−20	−30	−2	8.00	<0.001
	Motor	L	10	−8	−22	0	4.49	0.044
	Memory	R	139	24	−30	−2	10.08	<0.001

a Neurosynth term/function (www.neurosynth.org).

b Hemisphere: R, right, L, left.

c Cluster size; number of contiguous voxels with p<0.05.

d x, y, and z coordinates in MNI space.

e Familywise error corrected at the cluster level.

All results significant at *p*< .05, cluster-level family-wise error correction.

**Figure 4:**
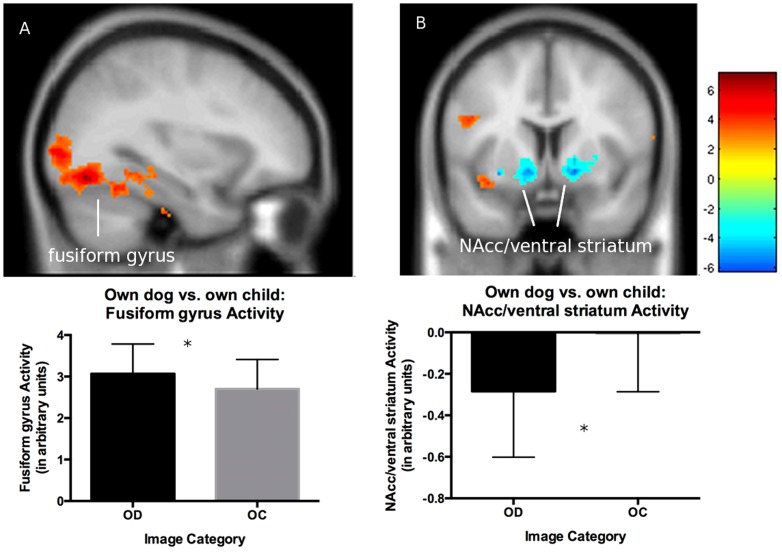
Brain activation maps and graphs for the own dog vs. own child contrast. There was greater activation for the own dog vs. own child contrast in fusiform gyrus (A) and less deactivation for own child vs. own dog contrast in NAcc/ventral striatum (B). Other conventions the same as in Figure 2.

There was greater activation in the own dog > own child contrast in a region involved in visual processing and social cognition (bilateral fusiform gyrus (Figure 4a): *t*(13)  = 5.29, *p* =  .036, cluster extent  = 114, MNI coordinates: x = −32, y = −70, z = −12; *t*(13)  = 5.25, *p* =  .043, cluster extent  = 106, MNI coordinates: x = −42, y = −80, z = −18; *t*(13)  = 5.57, *p* =  .001, cluster extent  = 308, MNI coordinates: x = 26, y = −76, z = −18) and less *deactivation* in regions associated with interoception (posterior insula: *t*(13)  = 3.97, *p* =  .025, cluster extent  = 130, MNI coordinates: x = −40, y = −14, z = 8) and social cognition (superior temporal gyrus: *t*(13)  = 4.46, *p* =  .019, cluster extent  = 179, MNI coordinates: x = 66, y = −16, z = 2). For the own child > own dog comparison, the only difference was less *deactivation* in a region involved in reward and affiliation (NAcc/ventral striatum (Figure 4b): *t*(13)  = 4.94, *p* =  .042, cluster extent  = 26, MNI coordinates: x = −10, y = 6, z = −8).

### Behavioral measures

**Figure 5:**
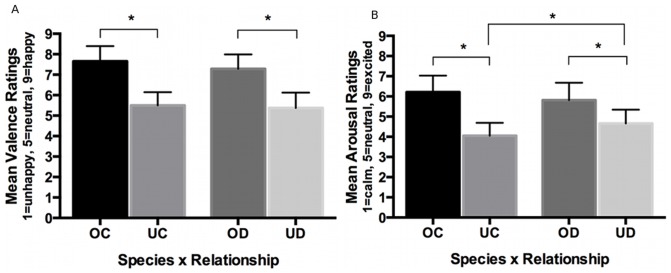
Graphs of the post-scan valence (Fig. 5a) and arousal (Fig. 5b) ratings show significant differences between own child (OC) > unfamiliar child (UC) and own dog (OD) > unfamiliar dog (UD; valence and arousal) and UD > UC (arousal). All *p*s < 0.05. Error bars depict 95% confidence intervals.

All participants reported that they loved and were attached to their dog, with mean LAPS scores of 48.6 of a possible 69. Thirteen of fourteen (93%) considered their dog a member of the family, and 13 felt very or extremely close to their dog, while one felt somewhat close. There was a main effect for relationship (own vs. unfamiliar) on valence [*F*(1,13)  = 53.14, *p*< .001] and arousal [*F*(1,13)  = 34.53, *p*< .001] with valence and arousal higher for own than other child and dog, and a relationship x species interaction for arousal [*F*(1,13)  = 8.85, *p* =  .011; Figure 5]. LAPS total score was correlated with mean valence ratings for the own dog images (*r*(12)  = 0.55, *p* =  .040) but not unfamiliar dog images (*p* =  .983). LAPS total score was not correlated with arousal ratings (*ps* > .7).

## Discussion

To our knowledge, this is the first report of a comparison of fMRI-related brain activation patterns in women when they viewed images of their child and dog. Here we report substantial overlap in brain activation patterns in regions involved in reward, emotion, and affiliation elicited by images of both a mother's own child and dog. These women also reported similar pleasantness (valence) and excitement (arousal) ratings for their child and dog with a larger difference in the own vs. unfamiliar child compared to the own vs. unfamiliar dog comparisons for arousal. Valence ratings of the own dog images were also positively correlated with self-reported pet attachment. Interestingly, images of their child activated the dopamine, oxytocin, and vasopressin-rich midbrain VTA/SNi, thought to be a critical brain region involved in reward and affiliation that was *not* activated by images of their dog. When viewing images of their own child, there was less deactivation in another key reward region (NAcc/ventral striatum) compared to viewing their own dog or an unknown child. It is important to note the ANOVA analysis resulted in a significant main effect of *relationship* (own vs. other), but no main effect of species or relationship x species interaction. However, the planned contrast of own child vs. own dog resulted in significant differences in several regions, including bilateral fusiform gyrus, posterior insula, superior temporal gyrus, and NAcc/ventral striatum. The discrepancy in the results from these two analyses may be explained by methodological differences in the ANOVA and the planned contrast (t-test) approaches. That is, the planned contrast tests whether there is a specific effect between two conditions (e.g., own child vs. own dog) while the ANOVA interaction tests whether there are *any* differences by relationship status (own or unfamiliar) at different levels of species (child or dog). Given the primary aim of the current study was to test the difference in mother's neural responses to their own child vs. own dog (not unfamiliar child vs. unfamiliar dog), the majority of the discussion has focused on these comparisons.

This report extends the mapping of the functional neuroanatomy of human relationships to an important human-animal relationship. A strength of the study is that it had a similar design to previous studies of brain response to visual images of familiar and unfamiliar people [42], friends and romantic partners to adults in love [22], [23], [43]–[47] and infants and children to mothers [23], [24], [37], [38], [48]–[50]; reviewed in [37], [51]. As observed in some of these prior studies of close human relationships, the amygdala, thought to be a critical region for bond formation, was activated to *both* the own child and dog images. The amygdala may be involved in providing the emotional tone and incentive salience that directs attention to the needs of the child and dog, which is critical for the formation of these pair bonds [24]. Another brain region critical to bond formation, the SNi/VTA, was *only* activated when mothers viewed images of their child. The SNi/VTA has a high density of dopamine, oxytocin, and vasopressin receptors that plays a critical role in reward-mediated attachment and affiliation [52], [53]. This replicates previous reports of maternal SNi/VTA activation to stimuli related to their child [23], [38], [54]. While SNi/VTA is also reported to have a critical function for other human-human relationships of evolutionary importance (romantic relationships; [22], [23]), this does not appear to extend to the human-pet bond [55], [56]. This could indicate that, in humans, the SNi/VTA is ‘central’ for the formation and maintenance of pair bonds that sustain and propagate our species.

There was also overlap in own child and own dog vs. fixation contrasts in brain areas associated with reward (mOFC, putamen; [37], [51], [57]), memory (hippocampus, thalamus; [37], [54], [58]), and visual/facial processing and social cognition (fusiform gyrus; [39], [49], [59]), which suggests importance for both the human-human and human-dog relationships.

We did not observe ventral striatum/NAcc activation in response to any of the visual stimulus categories. This is a critical node in the reward network, which may reinforce social interactions that lead to long-term pair bonds [24]. This finding is consistent with previous studies that reported no ventral striatum/NAcc activation when mother's viewed images of their older children or romantic partners [22], [23] but was activated to images of their infants [24], [60]. It is possible that the ventral striatum/NAcc is critical to the *formation* of pair bonds, while dorsal aspects of the striatum may be more crucial for the *maintenance* of these bonds. A similar transition from ventral to dorsal striatum driving behavior has been observed in the transition from voluntary to habitual behavior [61]. As in prior studies, we observed activation in other aspects of the striatum (putamen). We observed less *deactivation* in this ventral striatum/NAcc when mother's viewed images of their own child vs. both an unfamiliar child and their own dog, which may reflect less habituation [62].

While the fusiform gyrus was activated for both own child and dog images, there was greater magnitude and extent of activation in response to the own dog images when compared directly with the own child images. This region is central to visual and face processing and social cognition [39], [63]–[65]. Given the primacy of language for human-human communication, facial cues may be a more central communication device for dog-human interaction [66]. Face perception may contribute to the human-dog bond by helping owners identify their dog, use gaze direction to communicate, and interpret emotional states [65], [66].

### Caveats

Strengths of the study include the within-subjects design that allowed us to directly assess similarities and differences in response to the child and dog images with each participant serving as their own control, and a well-controlled image acquisition protocol which isolated the faces of dogs and children without including other features or contexts in the image that could complicate the interpretation of results if participants selected their own images from an existing set of photographs as previous studies have done. However, due to the cross-sectional nature of the design, it is not possible to determine whether the observed results relate to formation or maintenance of the pair bonds tested in this study. While we only included mothers who reported a healthy parenting relationship with their child, we did not strictly assess parent-child ‘attachment’ as traditionally defined and measured. We also studied a somewhat homogeneous group of mothers/pet owners: all women with young children between the ages of 2–10 and dogs that had been pets for 3–10.5 years. This homogeneity in ratings of attachment and emotional valence increased our power to detect effects of child vs. dog images on brain activation, but limited our ability to detect relationships between brain activation patterns and self-reported emotional ratings and attachment due to the restricted range of relationships. Due to scheduling constraints, we were unable to scan all women in the same menstrual phase, which has been shown to affect activation in reward-related brain areas [67]. Further research is needed to assess the generalizability of these findings to other relationships such as fathers, parents of adopted children, other animal species, and in mothers with a broader range of attachment.

## Summary and Conclusions

Mothers reported similar emotional ratings for their child and dog, which elicited greater positive emotional responses than unfamiliar children and dogs. While a common brain network involved in reward, emotion, and affiliation was activated when mothers viewed images of their child and dog, activation in the midbrain (VTA/SNi), a key brain region involved in reward and affiliation, characterized the response of mothers to images of their child and was not observed in response to images of their own dog. Mothers also had greater activation in the fusiform gyrus when viewing their own dog compared to when they viewed their own child. These results demonstrate that the mother-child and mother-dog bond share aspects of emotional experience and patterns of brain function, but there are also brain-behavior differences that may reflect the distinct evolutionary underpinning of these relationships.
